# Reductions in task positive neural systems occur with the passage of time and are associated with changes in ongoing thought

**DOI:** 10.1038/s41598-020-66698-z

**Published:** 2020-06-18

**Authors:** Adam Turnbull, Theodoros Karapanagiotidis, Hao-Ting Wang, Boris C. Bernhardt, Robert Leech, Daniel Margulies, Jonathan Schooler, Elizabeth Jefferies, Jonathan Smallwood

**Affiliations:** 1 Department of Psychology, University of York, York, UK; 2 Sackler Centre for Consciousness Science, University of Sussex, Brighton, United Kingdom; 3 Montreal Neurological Institute and Hospital, McGill University, Montreal, Canada; 4 Centre for Neuroimaging Science, Kings College, London, UK; 5 Centre National de la Recherche Scientifique (CNRS), Paris, France; 6 Psychological and Brain Sciences, University of California, Santa Barbara, USA

**Keywords:** Neuroscience, Cognitive neuroscience

## Abstract

Cognition is dynamic and involves both the maintenance of and transitions between neurocognitive states. While recent research has identified some of the neural systems involved in sustaining task states, it is less well understood how intrinsic influences on cognition emerge over time. The current study uses fMRI and Multi-Dimensional Experience Sampling (MDES) to chart how cognition changes over time from moments in time when external attention was established. We found that the passage of time was associated with brain regions associated with external attention decreasing in activity over time. Comparing this pattern of activity to defined functional hierarchies of brain organization, we found that it could be best understood as movement away from systems involved in task performance. Moments where the participants described their thoughts as off-task showed a significant similarity to the task-negative end of the same hierarchy. Finally, the greater the similarity of a participant’s neural dynamics to this hierarchy the faster their rate of increasing off-task thought over time. These findings suggest topographical changes in neural processing that emerge over time and those seen during off-task thought can both be understood as a common shift away from neural motifs seen during complex task performance.

## Introduction

Cognitive states change with the passage of time, both in form and content^1^. In recent years, neuroscience has established neurocognitive systems that act to maintain patterns of cognition in a particular task state^2–4^. However, experience such as mind-wandering^5–7^ suggest there are also intrinsic influences on these dynamics^8,9^. One barrier to understanding self-generated influences on the dynamics of our thoughts is the lack of an empirical framework for understanding the mechanisms for transitions between neurocognitive states with different types of mental content. The current study examined whether naturally occurring changes in ongoing thought are rooted in macroscopic functional cortical organisation.

Contemporary cognitive neuroscience has established that neural functioning is organised along multiple hierarchies that together are assumed to give rise to the structure of human cognition^10^. These hierarchies reflect different aspects of cognition including distinctions between unimodal and transmodal systems^11^, dissociations between sensory systems^12^, and neurocognitive patterns linked to complex task performance^2,13^. These hierarchies govern the topography of neural activity^2,12,14,15^ and our study tested whether this constraint extends to transitions between different cognitive states, and in particular the dynamics of off-task thinking. Off-task experiences are common in daily life, suggesting they are an important feature of human cognition^16,17^, and prior laboratory studies have shown that they increase with the passage of time^18–20^.

In the current study we built on these findings to focus on the dynamics of off-task thought, which can be captured in a straight forward manner using multi dimensional experience sampling (MDES). This method requires participants to provide intermittent descriptions of the contents of ongoing thought along multiple dimensions^21^. Recent work has used this method to reveal both the similarities and differences in the patterns that of ongoing thought take in the lab and in daily life^22^. Importantly in the context of this project, laboratory studies using MDES have captured a pattern off-task thought that increases with the passage of time^17–20^, suggesting that this approach provides a viable subjective window into an important dynamic feature of human cognition. Finally, prior studies that have combined experience sampling during tasks with measures of neural function have highlighted activity in multiple large scale systems during periods of off task thought. These networks include the default mode network^23–26^ as well as regions linked to executive control^24,27^.

In our study, participants performed a simple cognitive task while neural activity was measured using functional magnetic resonance imaging (fMRI). Multi-Dimensional Experience Sampling (MDES^6^) was used to measure moment-to-moment fluctuations in individuals’ experiences. We used these data to derive descriptions of the neural topography associated with the passage of time, and during off-task thought. We selected three well-established neuro-cognitive hierarchies reflecting (i) the distinction between unimodal and transmodal systems, (ii) visual and sensorimotor systems, and (iii) the patterns describing the brains response to cognitive tasks, identified in a previous study^11^. Using these spatial maps as descriptions of the constraints neural hierarchies place on function, we tested whether they provide a framework to understand the dynamic changes that characterise transitions between states of external task focus and off-task self-generated experiences. If neural hierarchies constrain the temporal dynamics linked to off-task thinking then (a) neural changes associated with the passage of time should match the topographical motifs seen in one or more neurocognitive hierarchies, (b) the same topographical neural motifs will be seen during patterns of off-task thought, and (c) the extent of these temporal changes should be related to individual variation in patterns of ongoing thought.

## Results and Discussion

### Brain regions changing with the passage of time

**Figure 1:**
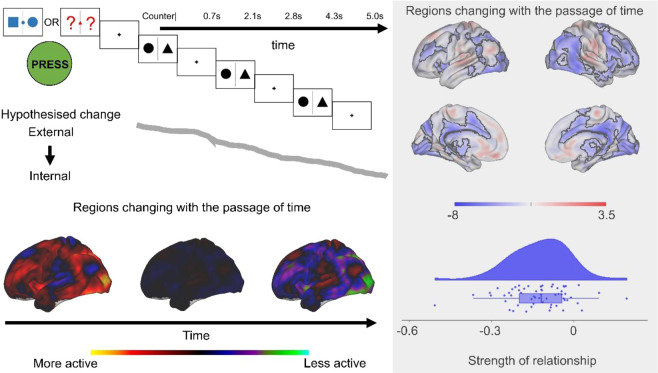
Brain regions change over time following task performance. This analysis looked for significant relationships between brain activity and the passage of time following a task event (target or thought probe: top right). This was based on the idea that external attention is established during external behaviour and then becomes subject to intrinsic influences with the passage of time while the participant remains uninterrupted. We found several large clusters of activity that showed a pattern of significantly decreasing activity with each non-target trial after the performance of an action (left). The global change in activity over time is represented by the unthresholded images in the bottom left. Neural results in the right hand panel are corrected at a cluster forming threshold of Z > 3.1, FWE-corrected p < 0.05.

We used a task in which people intermittently make behavioural responses to nominated target stimuli. It had two conditions, one requiring continuous monitoring (1-back) and another that did not (0-back, See top left hand panel of Fig. 1 and Fig. 5). In this context, we assume patterns of externally focused task-relevant cognition are momentarily established when participants make a response in the task. We used these moments as a reference point from which temporal changes can be calculated, generating a regressor describing for each non-target the amount of time passed since the last behavioural response (range = 2.1–15.8 s). Using this as an explanatory variable in a whole-brain analysis we found no regions that increased over time, however, multiple regions showed the reverse pattern (see Fig. 1, and Supplementary Table 2). Regions that decreased activity over time included parts of visual and attention networks. Meta-analytic decoding^28^ confirmed these regions are involved in external task performance (see Fig. 1).

### Gradient similarity of elapsed time

**Figure 2:**
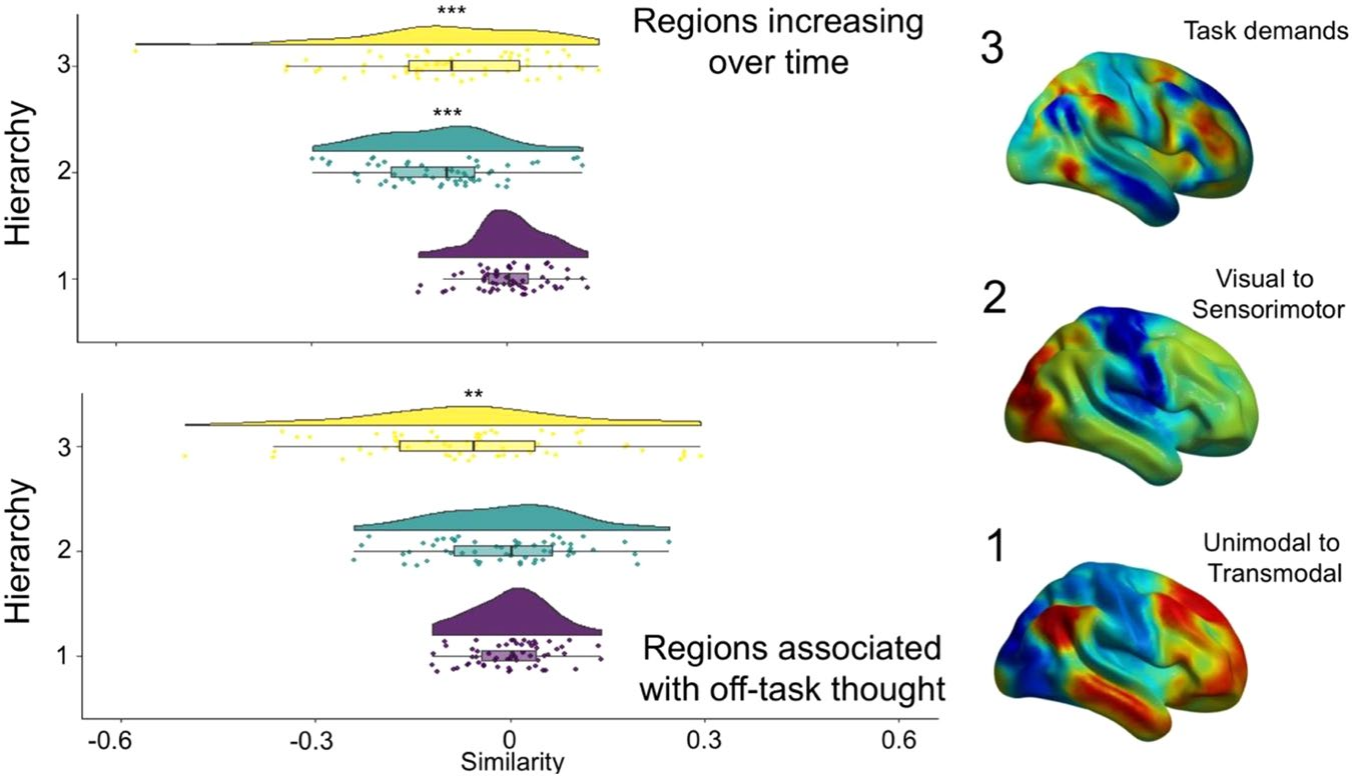
The brain dynamics associated with the passage of time can be understood as changes along neurocognitive functional hierarchies. The maps representing each participants’ neural correlation with time were compared to previously defined functional hierarchies^11^. Brain regions that changed over time were significantly related to the hierarchies representing task-demands as well as modality (top). When participants said they were having off-task thoughts neural patterns were similar to patterns seen in the absence of external demands (bottom).

Having documented how neural responses following task events change with time, we next examined whether the global topography of this pattern relates to either of the three neural hierarchies identified in our prior analyses^11^ (Fig. 2). After extracting the similarity of each participants’ elapsed time effects to each hierarchy (see Methods and Fig. 2), a series of one-sample t-tests determined neural patterns emerging with the passage of time show a significant association to (i) the modality gradient (t(58)=−8.457, p < 0.001) and (ii) the gradient highlighting the brain’s response to a task (t(58)=−4.878, p < 0.001). There was no association with the transmodal gradient (t(58)=0.395, p = 0.694). In our task, therefore, the effect of time on neural activity can be effectively characterised as neural changes along two functional hierarchies - away from visual processing and regions implicated in task-positive processes.

### Relationship between task-related gradient similarity and off-task thought

**Figure 3:**
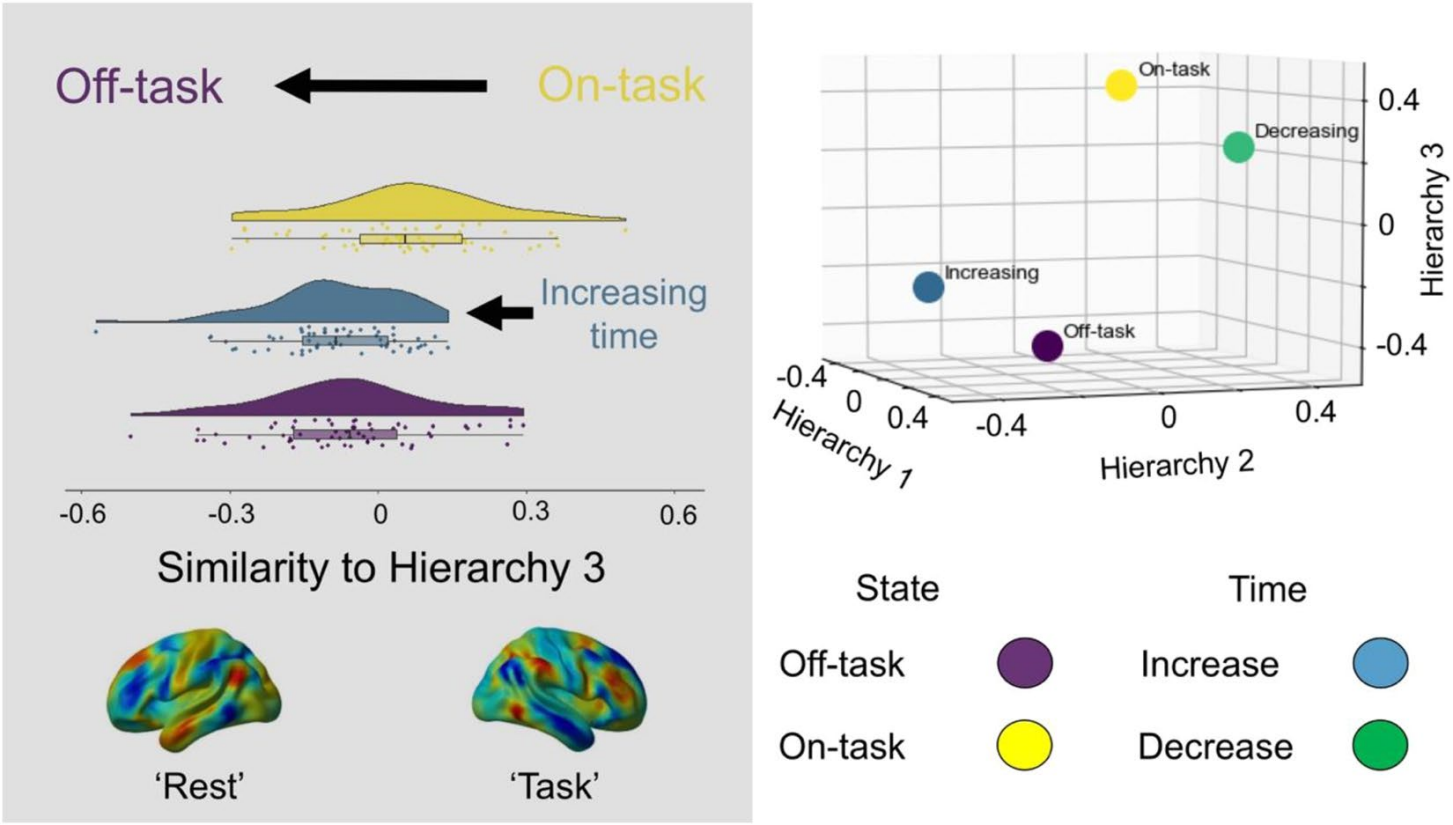
Neural dynamics associated with the passage of time and differences between on- and off-task thought are associated with common reductions in task positive regions. Brain activity during on-task thought looks similar to the task-positive end of the task-related gradient, as do regions that decrease over time. This suggests that over time a neural motif emerges that is similar to that seen during off-task thought, and mimics the patterns seen in the absence of a task (left). These same maps can be represented in a three-dimensional space based on each hierarchy (top right). In this plot it can be seen that neural patterns that decline over time (green), and patterns seen during on task states (yellow), are located in the top right quadrant. In contrast, patterns that increase over time (blue) and those associated with off task thought (purple) are located in the bottom right quadrant. Note that the values in the 3D plot are r-values from the group level results (including motion as a covariate) and are therefore related to, but not the same as, the beta-weights representing each subject’s similarity score displayed in the raincloud plots^29^.

We next examined whether neural changes emerging over time are similar to the topographical patterns seen during off-task thought. We used spatial maps describing patterns of off-task thought derived from this data set and published in our prior paper^27^. A simple correlation between the off-task map and the elapsed time map found that these were similar (r = 0.46). Next, using a similar approach as in our prior analyses (see Methods), we compared each participants’ brain activity during off-task thought to each of the three neurocognitive hierarchies (Fig. 2). This revealed the off-task spatial map shows a similar topography only to the task-related gradient (Gradient 3: t(58) = −2.944, p = 0.006; Gradient 1: t(58)=0.100, p = 0.921; Gradient 2: t(58) = −0.678, p = 0.500). To formally understand this similarity, we tested the distributions of off-task thought and regions that increased over time in terms of their similarity to the task-related gradient, and found no statistical evidence of a difference (D = 0.644, p = 0.801, see Methods). This establishes that patterns observed with the passage of time, and during off-task states, have a similar neural topography that entails reductions in neural motifs that reflect the brains’ response to tasks. All of these relationships are represented in the 3D scatter plot in the top right panel of Fig. 3.

### Individual variation in elapsed time and changes in off-task thought

**Figure 4:**
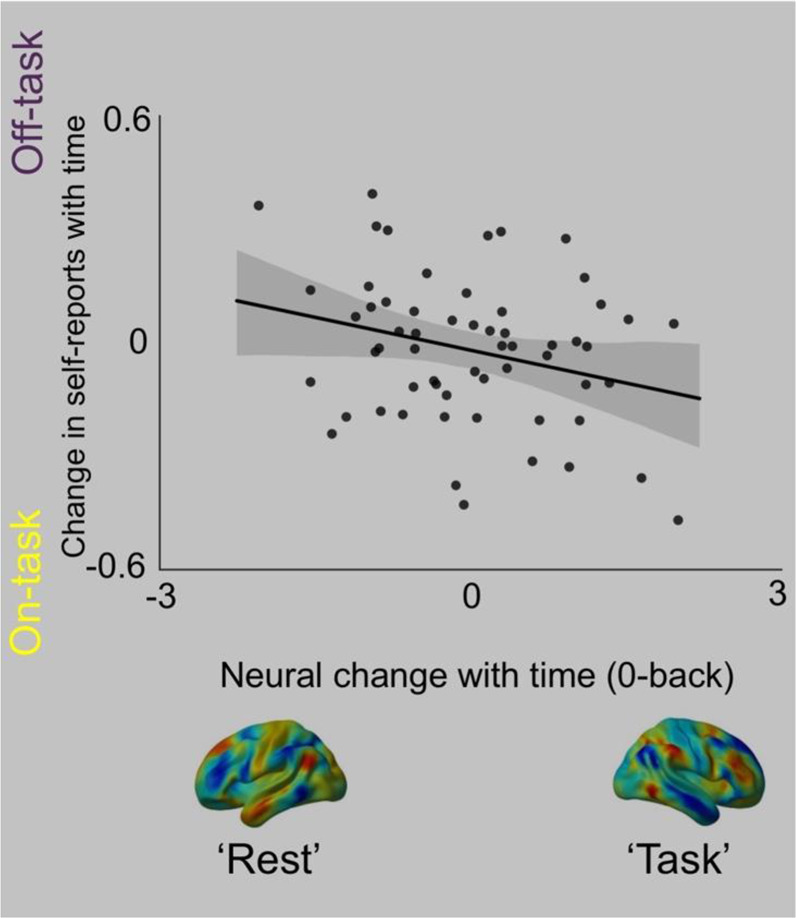
Greater neural shift towards task-negative regions during the 0-back task are associated with greater off-task thought. The horizontal axis of the scatter plot indicates the association between patterns of off-task thought and time. The vertical axis of the scatterplot indicates the change along the task positive hierarchy (Gradient 3) with the passage of time during the 0-back task. It can be seen that individuals who show the greatest shift away from task-positive regions during the 0 back task with time also show the strongest increase in off-task thought over time.

Finally, we examined whether neural changes that emerge over time have a direct association with the participants’ self-reports of off-task thought. We extracted the weightings of the off-task thought component from each probe and calculated for each individual how this changed with time (see Methods). We ran a repeated measures ANOVA in which the change along each neural hierarchy with time in each task were the explanatory variables and the change in off-task thought with time in each task were the outcome variables. This revealed a main effect of the change along hierarchy 3 in the 0-back task (F(1,49)=4.861, p = 0.032). Figure 4 plots the change in neural processing with time in the 0-back task against the average change in off-task thought over time. It can be seen in the scatter plot that those individuals who showed topographical changes in neural activity most strongly related to reductions in the patterns seen during active external task performance during the 0-back task, also showed the greatest overall shift to off-task thought with the passage of time. It is also clear that individuals who tended to maintain activity in task positive systems with time, tended to maintain better task focus. Together these analyses show that changes in the relative balance between task positive and task negative systems parrallel changes in participants self-reports of whether they were focused on the task or on other personally relevant information.

## Discussion

Together our study establishes topographical changes in neural processing that emerge over time and those seen during off-task thought can both be understood as a common shift away from neural motifs seen during complex task performance^2^. Our study used an experimental procedure that allows the passage of time to be calculated from moments when external focus is established, and so provides novel support for studies that use complex statistics to highlight the association between hidden neural dynamics and self-generated cognitive states^30–33^. It also complements work suggesting that the brain flexibly is temporarily reconfigured into states that support task performance^13^ by providing an understanding of how these states are disengaged over time following a task event. Previous studies have shown that the brain takes minutes to return to a stable baseline following task performance^34^. Our study provides a snapshot into the subjective correlates of these temporal dynamics, helping align neural and psychological perspectives.

Although our study highlights links between neural changes over time and the emergence of self-generated states, many important questions remain unanswered. First, our study highlighted a general pattern of how neural activity changed with time across both the 0-back and the 1-back task. In a previous study using trait-level variation in neural architecture^20^ we identified networks related to increasing off-task thought over time across both tasks. However, studies using self-reports highlight temporal dynamics in experience are often contextually bound; for example in this paradigm stronger increases in off-task thought with time are seen in the easier 0-back task in longer sessions outside the scanner^20^. Similarly, the results of this study identified that the overall degree to which an individual switched towards an off-task state with the passage of time was associated with neural changes away from patterns associated with task states over time in the 0-back task. More generally, it is clear that not all individuals show the same degree of neural changes away from task relevant material with the passage of time (see Fig. 4). It remains unclear, therefore, the degree to which temporal changes in neural activity are bound to specific task conditions, or reflect features of an individual that influence whether, and to what degree, their attention is likely to switch away from the task in hand. Our correlational design cannot address this question, so future work using transcranial magnetic stimulation or lesion paradigms in neurological patients may be needed to understand if causally disruptions to regions such as dorsolateral prefrontal cortex^27^ (DLPFC) or thalamus^13^ influence how neural processing changes with time. Additionally, it is also possible that increasing the difficulty gradient between the task conditions (e.g. by increasing memory load) would enable us to see differences in neural dynamics across the two tasks in the population as a whole.

Second, the observed topographical changes in neural function with time must be partly determined by the specific capacities that our tasks involve (e.g. attention, visual processing, and working memory). Accordingly, it is important to explore how time influences neural activity in contexts with richer semantic and sensory features (such as reading, listening to audiobooks, or watching movies). In these conditions prior work has highlighted regions within the default mode network as important in maintaining task focus^35^. It is possible that under these conditions regions of the default mode network may play a role in scaffolding attention over time because of their association with regions linked to semantic^36^ or episodic memory^37^.

Third, it is important to bear in mind that while our study highlights associations between patterns of off-task thought over time with neural dynamics that emerge over time, this association could emerge for many reasons. For example, motivation is known to play an important role in maintaining on task attention^38^, suggesting that part of the observed change in neural function may reflect relatively deliberate shift in the focus of ongoing thought. Finally, while our analysis highlights how changes in neural function are linked to patterns of off-task thought, this type of observation does not substantially constrain accounts of why time should lead to alternative patterns of thought^8^. In our prior study examining momentary differences in neural activity^27^, we found that a set of neural processes rooted in DLPFC, govern the regulation of off-task thought with respect to the current level of tasks demands. It is possible, that changes in neural activity over time reflects the hypothesised modulatory influence of the ventral attention, or salience, network on patterns of cognition^39–42^. Alternatively, brain systems focused on the thalamus may play an important role in orchestrating how neural processes change over time, given their documented role in organising task relevant neural processing^13^. Alternatively, it is possible that a pattern of task adaption in task positive regions that emerge with the passage of time could allow neural activity to shift away from the task relevant state. Thus, although our study establishes that temporal neural changes are at the core of how patterns of ongoing thoughts emerge over time, it leaves open the neural mechanism through which changes over time allows our minds the freedom to consider events other than those in our immediate environment.

## Methods

### Experimental model and participant details

#### Participants

A group of 63 young adults took part in a task-based fMRI study during which we used MDES during task performance to gain descriptions of experience at specific moments in time. After excluding participants (see Method Details) 59 participants (36 females, mean age=20.17 years, S.D. = 2.24 years) remained for data analysis. These data have been analysed before to understand the neural basis of off-task thought^27^. A subgroup of 34 participants were scanned twice for the data used in Sormaz *et al*.^14^. All participants were native English speakers, with normal/corrected vision, and no history of psychiatric or neurological illness. All participants were acquired from the undergraduate and postgraduate student body at the University of York. Both experiments were approved by the local ethics committee at both the York Neuroimaging Centre and the University of York’s Psychology Department. All volunteers gave informed written consent and were compensated in either cash or course credit for their participation. This experiment was carried out in accordance with the relevant guidelines and regulations.

### Method Details

#### Task performance

**Figure 5:**
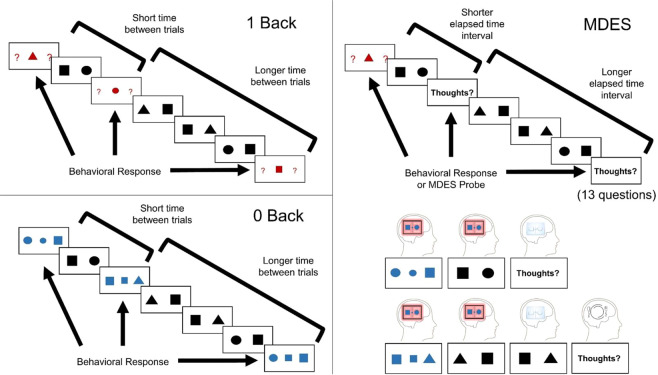
The task consisted of two conditions. In both conditions, participants had to make a spatial decision on the location of a shape shown in the middle of the target trial. In the 0-back, they had to say which side of the screen the shape was on during that trial (perceptually-guided decision). In the 1-back, they had to say which side of the screen the shape had been on the previous trial (memory-guided decision). On a quasi-random basis, the target trials were replaced by thought probes that asked 13 questions about the participants’ thoughts. These always started with a question about task focus, then the other 12 question (see Supplementary Table 2) were in a random order.

Task performance was identical to that used in the prior studies using this data^14,27^, and is similar to a task used in previous studies^20,43,44^. Experience was sampled in a task paradigm that alternated between blocks of 0-back and 1-back to manipulate attentional demands and working memory load (see Fig. 5). Non-target trials in both conditions were identical, consisting of black shapes (circles, squares, or triangles) separated by a line. In these trials the participant was not required to make a behavioural response. The shapes on either side of the line were always different. The colour of the centre line indicated to the participant the condition (0-back: blue, 1-back: red; mean presentation duration=1050 ms, 200 ms jitter). The condition at the beginning of each session was counterbalanced across participants.

During target trials, participants were required to make a behavioural response on the location of a specific shape. On these trials in the 0-back condition, a pair of shapes were presented (as in the non-target trials), but these were blue. Participants were instructed to indicate which shape (left or right) matched a small blue shape in the centre of the line down the middle of the screen. In the 1-back condition, the target trial consisted of two red question marks either side of the central line (in place of the shapes). There was a small shape in the centre of the screen as in the 0-back condition, but it was red. Participants had to indicate via button press which of the two shapes from the previous trial matched the central shape. Due to an error during data collection, reaction time was not measured during this task and accuracy was measured by the last button press during target trial presentation.

The contents of ongoing thought during this paradigm were measured using multi-dimensional experience sampling (MDES)0^6^. MDES probes occurred instead of a target trial on a quasi-random basis. When a probe occurred the participants were asked how much their thoughts were focused on the task, followed by 12 randomly shuffled questions about their thoughts (see Supplementary Table 1). All questions were rated on a scale of 1 to 4.

Each run was 9-minutes in length and there were four runs per scanning session. In each run, there was an average of six thought probes (three in each condition), so that there were on average 24 (SD = 3.30, mean=12 in each condition) MDES probes in each session.

### fMRI acquisition

fMRI acquisition follows a standard protocol used in studies in this laboratory, including those using the same data as this study^14,27^ and others^20^. MRI scanning was carried out at the York Neuroimaging Centre. Structural and functional data were acquired using a 3 T GE HDx Excite MRI scanner with an eight-channel phased array head coil tuned to 127.4 MHz. Structural MRI acquisition was based on a T1-weighted 3D fast spoiled gradient echo sequence (TR = 7.8 s, TE = minimum full, flip angle = 20°, matrix size = 256 × 256, 176 slices, voxel size = 1.13 × 1.13 × 1 mm). Functional data were recorded using single-shot 2D gradient echo planar imaging (TR = 3 s, TE = minimum full, flip angle = 90°, matrix size=64 × 64, 60 slices, voxel size = 3 mm isotropic, 180 volumes). A FLAIR scan with the same orientation as the functional scans was collected to improve coregistration between scans.

### Data pre-processing: fMRI

Data pre-processing was carried out in a similar way to previous studies using this data^14,27^. Two participants were excluded for falling asleep. Task-based functional and structural data were pre-processed and analysed using FMRIB’s Software Library (FSL version 4.1, http://fsl.fmrib.ox.ac.uk/fsl/fslwiki/FEAT/). Individual FLAIR and T1 weighted structural brain images were extracted using BET (Brain Extraction Tool). The functional data were pre-processed and analysed using the FMRI Expert Analysis Tool (FEAT). The individual subject analysis first involved motion correction using MCFLIRT and slice-timing correction using Fourier space time-series phase-shifting. After coregistration to the structural images, individual functional images were linearly registered to the MNI-152 template using FMRIB’s Linear Image Registration Tool (FLIRT). Functional images were spatial smoothed using a Gaussian kernel of FWHM 6 mm, followed by grand-mean intensity normalization of the entire 4D dataset by a single multiplicative factor, and highpass filtered (Gaussian-weighted least-squares straight line fitting, with sigma=100 s). Following pre-processing, the amount of participant head motion in each scan was inspected. Scans in which there was more than 1 mm absolute head motion or more than 0.2 mm relative head motion were discarded. Two participants were excluded due to having more than two runs removed. In total, three participants had one out of four runs removed due to motion and three participants had two out of four runs removed. Due to the possibility that motion could change over time following the motor action during external task performance, more stringent motion correction procedures were used than in previous studies using this data^14,27^.

### Quantification and statistical analysis

#### Principal component analysis

**Figure 6:**
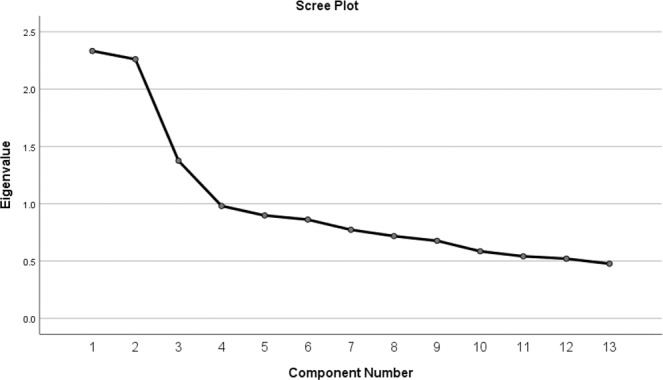
Four components were chosen from the screen plot following a PCA on the answers to 13 thought-related questions. For this study, only the first component (task-related thought) was used for analysis.

Behavioural analyses were carried out in SPSS (Version 24.0, 2016). The scores from the 13 mind wandering questions were entered into a principal component analysis (PCA) to describe the underlying structure of the participants’ responses. Following prior studies^14,20,27,43,44^ we concatenated the responses of each participant in each task into a single matrix and employed a PCA with varimax rotation. PCA was used in previous studies to establish the structure of ongoing thought across different conditions, and the same analysis has been used here to allow comparison with measures of off-task thought in those studies. We performed the same analysis with a rotation that does not induce orthogonality (oblimin), and found that none of the factors correlated above 0.25 suggesting that varimax rotation was appropriate^45^. Four components were selected based on the inflection point in the scree plot (see Fig. 6). These were defined (in order of decreasing eigenvalue) as Task-relatedness, Detail, Modality, and Emotion of thought based on their question loadings (Table 1). For the purposes of this study, only the task-related component was used for analysis.

**Table 1:** Factor loadings for the 13 questions used to assess ongoing thought on the 4 PCA factors. Only factor 1 was used in this study.

	Component			
	1	2	3	4
Deliberate	0.739	0.258	−0.134	−0.002
Detailed	0.350	0.569	−0.044	0.214
Emotion	0.381	−0.090	0.249	0.675
Evolving	0.145	0.533	−0.217	0.267
Focus	0.770	0.096	0.008	−0.033
Future	−0.219	0.334	0.017	0.511
Habit	−0.023	0.612	−0.025	0.060
Images	0.001	0.183	0.781	0.150
Other	−0.577	0.043	0.192	0.449
Past	−0.158	0.157	−0.146	0.538
Self	−0.571	0.369	0.017	0.034
Vivid	−0.080	0.719	0.244	−0.013
Words	0.160	0.152	−0.785	0.124

### Task-based fMRI analysis

Task-based analyses were carried out in FSL (FSL version 4.1, http://fsl.fmrib.ox.ac.uk/fsl/fslwiki/FEAT). The design files for this analysis can be found along with the rest of the details of the analyses in this paper at: https://github.com/adamgeorgeturnbull/elapsed_time. All explanatory variables (EVs) were convolved using FSL’s gamma function. EVs were modelled for the full duration of both non-target trials and correct target trials (incorrect trials were not included) in each condition separately. Any runs in which participants only made one correct response or fewer in either task were excluded. In total, four participants had one out of four runs removed, and one participant had two out of four runs removed. None of the participants who had runs excluded for poor task performance also had runs removed for motion. In addition to modelling the non-target and target trials, moderator EVs were included that modelled the length of the mini-block (since the last time the participant had to make a judgement during either a target trial or MDES probe). This was calculated by starting a counter at the end of the first non-target trial following a target event and adding the time for each non-target until either a target or MDES probe, in which case the counter was re-started. One moderator EV was added for time for the non-targets in each condition, and one for the targets. These EVs were mean-centred within each run. This gave a total of 8 EVs: four simple boxcar functions (two per condition for both non-targets and correct targets) and four moderators for these same time periods with mean-centred time since last event (see the right side of Fig. 1). A range of contrasts were included for a full description of the results. The contrasts of interest were a positive correlation with time across both tasks, a negative correlation with time across both tasks, and contrasts between the two tasks (regions significantly more related to time in one condition than the other) for targets and non-targets. The significant clusters can be seen in Supplementary Table 2. Fixed level analyses were performed for each participants included runs to provide an average brain response per person. Group level analyses were carried out using a cluster-forming threshold of Z > 3.1 and a whole-brain correction at p < 0.05 FWE-corrected^46^. Average framewise displacement was included at the group level to additionally control for effects relating to this nuisance variable. Figures were made using Connectome Workbench.

### Gradient similarity analysis

To assess to what extent the results of this fMRI experiment were occurring in the gradient space defined by Margulies *et al*.^11^, we performed a gradient similarity comparison. For each participant’s run-averaged brain response (fixed level output whole-brain zstat map) for significant contrasts of interest, we performed a spatial correlation using fsl_glm (demeaned using –demean) within a cortical mask defined by the regions included in the gradient. This gave three beta weights for each person representing how similar their brain response for each contrast was to each of the first three gradients defined by Margulies *et al*.^11^. To understand how the brain’s dynamics during task performance related to on- and off-task thought, this analysis was also carried out on the individual subject average brain responses for on- and off-task thought calculated in Turnbull *et al*.^27^. One-sample t-tests were carried out on these beta-weights to assess whether the group similarity of these responses was significantly related to any of the three gradients. These were Bonferroni corrected for multiple comparisons (alpha = 0.008). To directly compare the distributions of off-task thought and dynamic changes in terms of their relationship to the task-related gradient, we performed a Kolmogorov–Smirnov test in SPSS (see Results). Figures were made in R using raincloud plots^29^.

### Relationship to changes in off-task thought

To examine whether there was a relationship between the changes in the brain over time during task performance in gradient space and the change in off-task thought, we extracted the counter score (representing the length of time since the last response event) for each MDES probe. The time since the last target or preceding thought probe was correlated with off-task thought within each participant in each condition. These values were Fisher z-transformed. There was negligible average change in thoughts over time (mean=0.0194, S.D. = 0.19, t(58) = 0.765, p = 0.447). Studies with longer time periods suggest that off-task thoughts increase over time in a way that relates to individual differences in cognitive performance^18,20^. The time periods here may be too short to identify these changes on a group level, however, the relationships that have been found to changes in neural function suggest they are sufficient to identify meaningful individual differences. We ran a repeated measures ANOVA in which the change along each neural hierarchy with time in each task were the dependent variables. The similarity of the neural elapsed time effects for non-targets to each of the three first gradients from Magulies *et al*.^11^ in each task were entered as covariates of interest. Framewise displacement, age, and gender were entered as covariates of no interest. Data were mean-centered before performing this analysis.

## Supplementary information

Supplementary Information.
