# Author Correction: Reductions in task positive neural systems occur with the passage of time and are associated with changes in ongoing thought

**DOI:** 10.1038/s41598-020-69654-z

**Published:** 2020-07-28

**Authors:** Adam Turnbull, Theodoros Karapanagiotidis, Hao-Ting Wang, Boris C. Bernhardt, Robert Leech, Daniel Margulies, Jonathan Schooler, Elizabeth Jefferies, Jonathan Smallwood

**Affiliations:** 1 Department of Psychology, University of York, York, UK; 2 Sackler Centre for Consciousness Science, University of Sussex, Brighton, UK; 3 Montreal Neurological Institute and Hospital, McGill University, Montreal, Canada; 4 Centre for Neuroimaging Science, Kings College, London, UK; 5 Centre National de La Recherche Scientifique (CNRS), Paris, France; 6 Psychological and Brain Sciences, University of California, Santa Barbara, USA

Correction to: *Scientific Reports* https://doi.org/10.1038/s41598-020-66698-z, published online 18 June 2020

The Acknowledgements section in this Article was omitted. The Acknowledgements section should read:

“This project was supported by European Research Council Consolidator awarded to JS (WANDERINGMINDS – 646927).”

