# Supplementary material for: Reductions in task positive neural systems occur with the passage of time and are associated with changes in ongoing thought
Source: Sci Rep. 2020 Jun 18;10:9912. doi: 10.1038/s41598-020-66698-z (PMC7303126; doi:10.1038/s41598-020-66698-z)
Supplement: Supplementary file 1 — Supplementary Information. [file 41598_2020_66698_MOESM1_ESM.docx]

Slip-sliding away: associated reductions in task positive neural systems emerge with the passage of time and during off-task thought

Adam Turnbull^1^, Theodoros Karapanagiotidis^1^, Hao-Ting Wang^2^, Boris Bernhardt^3^, Robert Leech^4^, Daniel Margulies^5^, Jonathan Schooler^6^, Elizabeth Jefferies^1^, Jonathan Smallwood^1^.

Corresponding author: Adam Turnbull, email: agt520@york.ac.uk.

^1^Department of Psychology, University of York, York, UK

^2^Sackler Centre for Consciousness Science, University of Sussex, Brighton, United Kingdom.

^3^Montreal Neurological Institute and Hospital, McGill University, Montreal, Canada.

^4^Centre for Neuroimaging Science, Kings College, London, UK.

^5^Centre National de la Recherche Scientifique (CNRS), Paris, France.

^6^Psychological and Brain Sciences, University of California, Santa Barbara, USA

This project was supported by European Research Council Consolidator awarded to JS (WANDERINGMINDS – 646927).

**Supplementary Materials**

| Dimensions | Questions | 1 | 4 |
| --- | --- | --- | --- |
| Task | My thoughts were focused on the task I was performing. | Not at all | Completely |
| Future | My thoughts involved future events. | Not at all | Completely |
| Past | My thoughts involved past events. | Not at all | Completely |
| Self | My thoughts involved myself. | Not at all | Completely |
| Person | My thoughts involved other people. | Not at all | Completely |
| Emotion | The content of my thoughts was: | Negative | Positive |
| Images | My thoughts were in the form of images. | Not at all | Completely |
| Words | My thoughts were in the form of words. | Not at all | Completely |
| Vivid | My thoughts were vivid as if I was there. | Not at all | Completely |
| Detailed | My thoughts were detailed and specific. | Not at all | Completely |
| Habit | This thought has recurrent themes similar to those I have had before. | Not at all | Completely |
| Evolving | My thoughts tended to evolve in a series of steps. | Not at all | Completely |
| Deliberate | My thoughts were: | Spontaneous | Deliberate |

Table S1. Mind wandering questions asked to each participant during MDES. The first question was always “Task” then the other 12 questions in a random order. The scores from these questions were entered into a PCA.

| **Trial type** | **Relationship to time** | **Cluster size** | **p-value** | **MNI coordinates** |
| --- | --- | --- | --- | --- |
| **Non-target** | **Negative** | **33357**  **359** | **<.0001**  **<.0001** | **(12, -74, 34)**  **(-30, 38, 32)** |
| Target | Negative | 192 | .0311 | (4,46,-8) |

Table S2. Description of the significant clusters produced by the contrasts of interest after correction at a cluster forming threshold of Z>3.1, p>.05 FWE-corrected.
